# Synthesis of an Activatable Tetra-Substituted Nickel Phthalocyanines-4(3H)-quinazolinone Conjugate and Its Antibacterial Activity

**DOI:** 10.1155/2019/5964687

**Published:** 2019-04-17

**Authors:** Asma M. Elsharif, Tamer E. Youssef, Suhailah S. Al-Jameel, Hanan H. Mohamed, Mohammad Azam Ansari, Suriya Rehman, Sultan Akhtar

**Affiliations:** ^1^Department of Chemistry, College of Science, Imam Abdulrahman Bin Faisal University, P.O. Box 1982, Dammam 31441, Saudi Arabia; ^2^Basic and Applied Scientific Research Center, Imam Abdulrahman Bin Faisal University, P.O. Box 1982, Dammam 31441, Saudi Arabia; ^3^Department of Epidemic Disease Research, Institute of Research and Medical Consultation (IRMC), Imam Abdulrahman Bin Faisal University, Dammam 31441, Saudi Arabia; ^4^Department of Physics Research, Institute of Research and Medical Consultation (IRMC), Imam Abdulrahman Bin Faisal University, Dammam 31441, Saudi Arabia

## Abstract

The aim of this study was to synthesize a series of nickel(II)phthalocyanines (NiPcs) bearing four 4(3H)-quinazolinone ring system units, (qz)_4_NiPcs **4a–d**. The electronic factors in the 4(3H)-quinazolinone moiety that attached to the NiPc skeleton had a magnificent effect on the antibacterial activity of the newly synthesized (qz)_4_NiPcs **4a–d** against *Escherichia coli*. The minimum MICs and MBCs value were recorded for compounds **4a**, **4b**, **4c**, and **4d**, respectively. The results indicated that the studied (qz)_4_NiPcs **4a–d** units possessed a broad spectrum of activity against *Escherichia coli*. Their antibacterial activities were found in the order of **4d** > **4c** > **4b** > **4a** against *Escherichia coli*, and the strongest antibacterial activity was achieved with compound **4d**.

## 1. Introduction

To date, a great variety of phthalocyanines (Pcs) derivatives functionalized with substituted heterocycles such as pyridyloxy, 4-pyridylmethyloxy, and *N*-methyl morpholiniumethoxy substituents have received attention as antibacterial agents [1–3]. The antimicrobial properties of various derivatives of phthalocyanines such as zinc phthalocyanine-silver nanoparticle conjugates [4], octacationic zinc phthalocyanines bearing 1,2-ethanediamine groups and the quaternized derivatives [5], pentalysine *β*-carbonylphthalocyanine zinc [6], and silicon phthalocyanine [7] against Gram-negative and Gram-positive bacteria and biofilm-forming bacteria have been reported by the researchers. 4(3H)-Quinazolinones are known to possess interesting drugs with diverse biological activities. They were used to modify the biological properties of several other compounds. The major effective biological activities and pharmacological properties of their derivatives include analgesic [8], anticonvulsant [9], antidiabetic [10, 11], antitubercular and antibacterial effects [12], antihypertensive [13], antiviral [14], and cancer chemotherapy [15].

Earlier, Youssef and Hanack have described novel symmetrically and asymmetrically NiPcs bearing heterocyclic moieties for pharmaceutical application [16], in connection with a previous work and our current interest in the synthesis of Pcs derivatives functionalized with substituted heterocycles for biological evaluations [17, 18]. We described herein a facile convenient synthesis of novel tetra-substituted nickel phthalocyanines based on the heterocyclic moiety (i.e., 4(3H)-quinazolinone ring system (qz)_4_NiPcs (**4a–d**)). However, antibacterial properties of tetra-substituted 4(3H)-quinazolinone nickel(II)phthalocyanine derivatives have not been yet explored. To the best of our knowledge, this is the first report which aims to modify the structural activity of nickel(II)phthalocyanines conjugate with four 4(3H)-quinazolinone units and evaluate their parameters required for the structure-function relationship for antibacterial properties. The antibacterial activity results obtained for the newly formed (qz)_4_NiPcs **4a–d** show promising antibacterial properties against *Escherichia coli*.

## 2. Materials and Methods

All reagents and solvents were used without further purification. 4-Nitrophthalonitrile, 2-methyl-4(3H)-quinazolinone, 2-phenyl-4(3H)-quinazolinone, 2-(trifluoromethyl)-4(3H)-quinazolinone, and 2-mercapto-4(3H)-quinazolinone were purchased commercially from Aldrich and used as received. All solvents (GR grade) from Merck (Darmstadt, Germany) were distilled. Silica gel thin-layer chromatography (TLC) plates of 250 microns from Analtech (Newark, DE, USA) were used.

Melting points were uncorrected and determined by the open capillary method. IR spectra were recorded on a Nicolet Magna 560 spectrophotometer with spectral range 4000–400 cm^−1^. ^1^H-NMR spectra were recorded in dimethyl sulfoxide (DMSO) using a BVT 3000 Bruker Spectro spin instrument operating at 300.13 MHz. UV-Vis spectra were recorded in dimethyl formamide (DMF) using an Agilent 8453 UV-Vis spectrophotometer. Field depolarization mass spectroscopy technique (FDMS) mass spectra were recorded using a Varian MAT 711A spectrometer and reported in mass/charge (*m*/*z*). The electron ionization technique (EIMS) operated at 70 eV and reported in mass/charge (*m*/*z*). The Carlo Erba elemental analyzer 1106 was used to perform elementary analyses. Thin layer chromatography (TLC) on precoated silica gel plates was used to determine the purity of all synthesized compounds utilizing different eluents with different ratios as developing solvent systems. The morphological features of as-synthesized tetra-substituted 4(3H)-quinazolinone nickel(II)phthalocyanine derivatives were examined before and after the bacteria treatment by scanning electron microscopy (SEM). The synthesized powder was also examined by transmission electron microscopy (TEM) in order to analyze the structure of the material with high resolution. SEM (FEI, ISPECT S50, Czech Republic) was operated at 20 kV, and TEM (FEI, Morgagni, Czech Republic) was performed at 80 kV. The electronic micrographs were recorded at 30 kx (SEM) and 180 kx (TEM) magnifications to obtain the representative features of the specimens. For SEM, the samples were mounted on a metallic stub with a double-sided adhesive tape and applied a thin layer of gold using sputter coating machine (Quorum, Q150R ES, UK) to minimize the less conductive nature of the material from charging. For TEM, powder was dispersed in ethanol, sonicated for 5–7 minutes, and deposited onto TEM grid. TEM grids were air-dried and mounted into the TEM.

### 2.1. Typical Procedure for Synthesis of 4(3H)-Quinazolinone-phthalonitrile Precursors **3a–d**

A mixture of 4-nitrophthalonitrile **1** (4.4 mg, 2.6 mmol) and quinazolinone derivatives **2a–d,** (4.4 mg, 2.75 mmol) **2a**, (5.7 mg, 2.5 mmol) **2b**, (4.2 mg, 2.4 mmol) **2c**, (4.3 mg, 2.4 mmol) **2d,** was dissolved in dry DMF (70 mL) and then stirred for 40 min at room temperature. A finely grounded K_2_CO_3_ (excess) was added portionwise over 4 hours and then stirred for 24 h at 75–80°C. The mixture was cooled to room temperature. It was poured into ice water. The crude product was held at 2.5 h and filtered off, and the mixture washed with water and dried under vacuum. The crude products were purified by column chromatography (silica gel, dichloromethane/*n*-hexane) in different ratios (9 : 1/8 : 2 v/v), yielding 5.5 mg (78%) of the pure phthalonitrile **3a**, 6.1 mg (70%) of **3b**, 5.0 mg (69%) of **3c**, and 4.8 mg (70%) of **3d**.

#### 2.1.1. Synthesis of 2-Methyl-4(3H)-quinazolinone-phthalonitrile **3a**

Prepared from 2-methyl-4(3H)-quinazolinone (**2a**) as a white solid; m.p. 291–292°C; IR (KBr): *v* = 3071–3069 (Ar-H_str_), 2966, 2869 (C-H_str_, CH_3_), 2233 (CN_str_), 1678 (C=O_str_, qz ring), 1658 (C-N; C-C); 1586, 1570, 1480 (C–CH); 1421 mPh, 1419, 855, 742, 746 d(C–C), 644, 521 cm^−1^. ^1^H-NMR (DMSO-*d*_6_): *δ* = 1.55 (3H, s, CH_3_-qz), 7.7–7.9 (4H, m, Ar-H, quinazolinone moiety), 8.24 (1H, dd, 5-H), 8.38 (1H, d, 6-H), 8.40 (1H, s, 3-H) ppm. MS (EI): *m*/*z* = 286.29 (M^+^). Elemental analysis: C_17_H_10_N_4_O found C 70.97, H 3.19, N 19.11, Calcd. C 71.32, H 3.52, N 19.57.

#### 2.1.2. Synthesis of 2-Phenyl-4(3H)-quinazolinone-phthalonitrile **3b**

Prepared from 2-phenyl-4(3H)-quinazolinone (**2b**) as a white solid; m.p. 311–314°C; IR (KBr): *v* = 3071–3066 (Ar-H_str_), 2960, 2872 (C-H_str_, CH_3_), 2238 (CN_str_), 1680 (C=O_str_, qz ring), 1664 (C-N; C-C); 1581, 1575, 1483 (C–CH); 1429 mPh, 1421, 855, 740, 750 d(C–C), 640, 521 cm^−1^. ^1^H-NMR (DMSO-*d*_6_): *δ* = 7.2–7.5 (4H, m, Ar-H, quinazolinone moiety), 8.10 (1H, dd, 5-H), 8.26 (1H, d, 6-H), 8.37 (1H, s, 3-H), 8.55 (5H, m, ph-qz) ppm. MS (EI): *m*/*z* = 348.36 (M^+^). Elemental analysis: C_22_H_12_N_4_O, Found C 70.97, H 3.87, N 18.98, Calcd. C 71.32, H 3.52, N 19.57.

#### 2.1.3. Synthesis of 2-Trifluoromethyl-4(3H)-quinazolinone-phthalonitrile **3c**

Prepared from 2-trifluoromethyl-4(3H)-quinazolinone (**2c**), as a white solid; m.p. 270–272°C; IR (KBr): *v* = 3060–3068 (Ar-H_str_), 2970, 2874 (C-H_str_, CH_3_), 2590 (SH_str_), 2230 (CN_str_), 1677 (C=O_str_, qz ring), 1668 (C-N; C-C); 1584, 1578, 1470 (C–CH); 1427 mPh, 1412, 853, 745, 748 d(C–C), 646, 530 cm^−1^. ^1^H-NMR (DMSO-*d*_6_): *δ* = 7.3–7.6 (4H, m, Ar-H, quinazolinone moiety), 8.49 (1H, dd, 5-H), 8.56 (1H, d, 6-H), 8.68 (1H, s, 3-H) ppm. MS (EI): *m*/*z* = 340.26 (M^+^). Elemental analysis: C_17_H_7_N_4_OF_3_, Found C 60.61, H 2.57, N 14.71, Calcd. C 60.01, H 2.07, N 14.01.

#### 2.1.4. Synthesis of 2-Mercapto-4(3H)-quinazolinone-phthalonitrile **3d**

Prepared from 2-mercapto-4(3H)-quinazolinone (**2d**), as a white solid; m.p. 287–290°C; IR (KBr): *v* = 3066–3070 (Ar-H_str_), 2968, 2871 (C-H_str_, CH_3_), 2597 (SH_str_), 2230 (CN_str_), 1677 (C=O_str_, qz ring), 1666 (C-N; C-C); 1581, 1575, 1470 (C–CH); 1427 mPh, 1411, 851, 746, 743 d(C–C), 648, 530 cm^−1^. ^1^H-NMR (DMSO-*d*_6_): *δ* = 3.30 (1H, s, SH-qz), 7.3–7.6 (4H, m, Ar-H, quinazolinone moiety), 8.41 (1H, dd, 5-H), 8.54 (1H, d, 6-H), 8.64 (1H, s, 3-H) ppm. MS (EI): *m*/*z* = 304.33 (M^+^). Elemental analysis: C_16_H_8_N_4_OS, found C 62.87, H 2.07, N 19.11, Calcd. C 63.15, H 2.65, N 18.41.

### 2.2. Typical Procedure for Synthesis of Tetra [4(3H)-Quinazolinone]phthalocyaninatonickel(II), [(qz)_4_NiPcs] **(4a–d)**

A solution of 4(3H)-quinazolinone-phthalonitrile derivative **3a–d** (5.5 mg, 2.04 mmol) **3a**, (6.3 mg, 2.08 mmol) **3b**, (6.6 mg, 2.05 mmol) **3c**, (7.2 mg, 2.04 mmol) **3d** and nickel(II) acetate dihydrate (0.22 g, 0.09 mmol) in 15 mL of *n*-pentanol. The mixture was stirred for 20 min under argon atmosphere. DBU (6 mL, 0.07 mmol) was added, and the mixture was refluxed for 30 h at 140–145°C. It was cooled at room temperature and then precipitated with methanol (10 mL). The solid was filtered and then washed with water and dried under vacuum. The crude products were purified by column chromatography (silica gel, chloroform/*n*-hexane) in different ratios (9 : 1/8 : 2 v/v), yielding 18 mg (75%) of the pure NiPc **4a**, 21 mg (72%) of **4b**, 18 mg (73%) of **4c,** and 14 mg (70%) of **4d**.

#### 2.2.1. Synthesis of Tetra [2-Methyl-4(3H)-quinazolinone]phthalocyaninatonickel(II), [(Me-qz)_4_NiPc] (**4a**)

IR (KBr): *v* = 3074–3069 (Ar-H_str_), 2977, 2878 (C-H_str_, CH_3_), 1673 (C=O_str_, qz ring), 1660 (C-N; C-C); 1582, 1579, 1470 (C–CH); 1413 mPh, 1411, 858, 740, 748 d(C–C), 644, 528 cm^−1^. ^1^H-NMR (DMSO-*d*_6_): *δ* = 1.33–1.7 (12H, m, CH_3_-qz), 7.4–7.8 (16H, m, Ar-H, quinazolinone moiety), 8.40 (4H, dd, 5-H), 8.55 (4H, d, 6-H), 8.62 (4H, s, 3-H) ppm. UV-vis (DMF): *λ*_max_ (nm): 670, 625, 356 sh, 294 nm. MS (FD): *m*/*z* = 1203.84 (M^+^). Elemental analysis: C_68_H_40_N_16_O_4_Ni, Found C 68.01, H 3.87, N 18.97, Calcd. C 67.84, H 3.35, N 18.62.

#### 2.2.2. Synthesis of Tetra [2-Phenyl-4(3H)-quinazolinone]phthalocyaninatonickel(II), [(Ph-qz)_4_NiPc] (**4b**)

IR (KBr): *v* = 3074–3065 (Ar-H_str_), 2977, 2872 (C-H_str_, CH_3_), 1675 (C=O_str_, qz ring), 1653 (C-N; C-C); 1578, 1573, 1475 (C–CH); 1440 mPh, 1404, 852, 740, 745 d(C–C), 649, 525 cm^−1^. ^1^H-NMR (DMSO-*d*_6_): *δ* = 7.2–7.5 (16H, m, Ar-H, quinazolinone moiety), 8.03 (4H, dd, 5-H), 8.15 (4H, d, 6-H), 8.36 (4H, s, 3-H), 8.5–8.8 (20H, m, ph-qz) ppm. UV-vis (DMF): *λ*_max_ (nm): 682, 620, 352 sh, 258 nm. MS (FD): *m*/*z* = 1452.12 (M^+^). Elemental analysis: C_88_H_48_N_16_O_4_Ni, Found C 66.93, H 3.64, N 19.03, Calcd. C 72.79, H 3.33, N 15.43.

#### 2.2.3. Synthesis of Tetra [2-Trifluoromethyl-4(3H)-quinazolinone]phthalocyaninatonickel(II), [(CF_3_-qz)_4_NiPc] (**4c**)

IR (KBr): *v* = 3072–3070 (Ar-H_str_), 2974, 2872 (C-H_str_, CH_3_), 1680 (C=O_str_, qz ring), 1665 (C-N; C-C); 1578, 1578, 1479 (C–CH); 1442 mPh, 1410, 853, 745, 748 d(C–C), 649, 523 cm^−1^. ^1^H-NMR (DMSO-*d*_6_): *δ* = 7.4–7.7 (16H, m, Ar-H, quinazolinone moiety), 8.21 (4H, dd, 5-H), 8.36 (4H, d, 6-H), 8.56 (4H, s, 3-H) ppm. UV-vis (DMF): *λ*_max_ (nm): 688, 614, 330 sh, 290 nm. MS (FD): *m*/*z* = 1419.73 (M^+^). Elemental analysis: C_68_H_28_N_16_O_4_F_12_Ni, Found C 57.01, H 2.05, N 16.08, Calcd. C 57.53, H 1.99, N 15.79.

#### 2.2.4. Synthesis of Tetra [2-Mercapto-4(3H)-quinazolinone]phthalocyaninatonickel(II), [(SH-qz)_4_NiPc] (**4d**)

IR (KBr): *v* = 3072–3070 (Ar-H_str_), 2974, 2875 (C-H_str_, CH_3_), 1680 (C=O_str_, qz ring), 1654 (C-N; C-C); 1578, 1573, 1478 (C–CH); 1442 mPh, 1403, 855, 742, 749 d(C–C), 648, 524 cm^−1^. ^1^H-NMR (DMSO-*d*_6_): *δ* = 3.40 (4H, s, SH-qz), 7.4–7.7 (16H, m, Ar-H, quinazolinone moiety), 8.30 (4H, dd, 5-H), 8.40 (4H, d, 6-H), 8.62 (4H, s, 3-H) ppm. UV–vis (DMF): *λ*_max_ (nm): 690, 618, 335 sh, 292 nm. MS (FD): *m*/*z* = 1276 (M^+^). Elemental analysis: C_64_H_32_N_16_O_4_S_4_Ni, Found C 59.06, H 2.06, N 16.97, Calcd. C 60.42, H 2.53, N 17.56.

### 2.3. Characterization of Antibacterial Activity of Tetra-Substituted 4(3H)-Quinazolinone Nickel(II)phthalocyanine Derivatives

*Escherichia coli* was chosen to investigate the antibacterial properties of tetra-substituted 4(3H)-quinazolinone nickel(II)phthalocyanine derivatives, and then *E. coli* cultures were grown overnight in nutrient broth in a shaking incubator (200 rpm) at 37°C. Its bacterial culture was then washed 2-3 times with phosphate-buffered saline. The *Escherichia coli* suspensions were diluted with sterile 0.9% NaCl solution to reach concentrations of approximately 10^7^ CFU/ml.

### 2.4. Minimal Inhibitory Concentration (MIC)

The antibacterial activity of tetra-substituted 4(3H)-quinazolinone nickel(II)phthalocyanine derivatives was assessed using the standard agar dilution method [19, 20]. The MIC was determined on MHA plates using serial dilutions of tetra-substituted 4(3H)-quinazolinone nickel(II)phthalocyanine derivatives in concentration range from 32 to 1 mg/ml. The MIC is the lowest concentration of compounds at which no visible growth of the bacteria was seen [19, 20].

### 2.5. Minimal Bactericidal Concentration (MBC)

The MIC plates which have no growth were further selected for MBC assessment [20]. Then, 100 *μ*l 0.9% normal saline was added onto the MIC plates and then transferred to another freshly prepared MHA plate without supplementing with tested compounds. They incubated it at 37°C for 24 h [20]. The lowest concentration of tetra-substituted 4(3H)-quinazolinone nickel(II)phthalocyanine derivatives at which no growth of bacterial cells has been found or less than three CFUs have been present were recorded as MBC [19, 20].

### 2.6. Effect of Tetra-Substituted 4(3H)-Quinazolinone Nickel(II)phthalocyanine Derivatives on the Morphology of *Escherichia coli*

Further, the effects of tetra-substituted 4(3H)-quinazolinone nickel(II)phthalocyanine derivatives on the morphology of *Escherichia coli* cells were investigated by scanning electron microscope as previously reported [20]. Briefly, ∼10^6^ CFU/ml of *Escherichia coli* cells treated with tetra-substituted 4(3H)-quinazolinone nickel(II)phthalocyanine derivatives was incubated at 37°C overnight. After incubation, the treated and untreated bacterial cells were centrifuged at 12000 rpm for 10 min. The pellets were washed with PBS and fixed with 2.5% glutaraldehyde followed by 1% osmium tetroxide. After washing, samples were dehydrated by a series of ethanol [20]. The cells were fixed on the aluminum stubs, then dried in a desecrator, and coated with gold. Finally, samples were examined at an accelerating voltage of 20 kV by SEM.

## 3. Results

**Figure 1:**
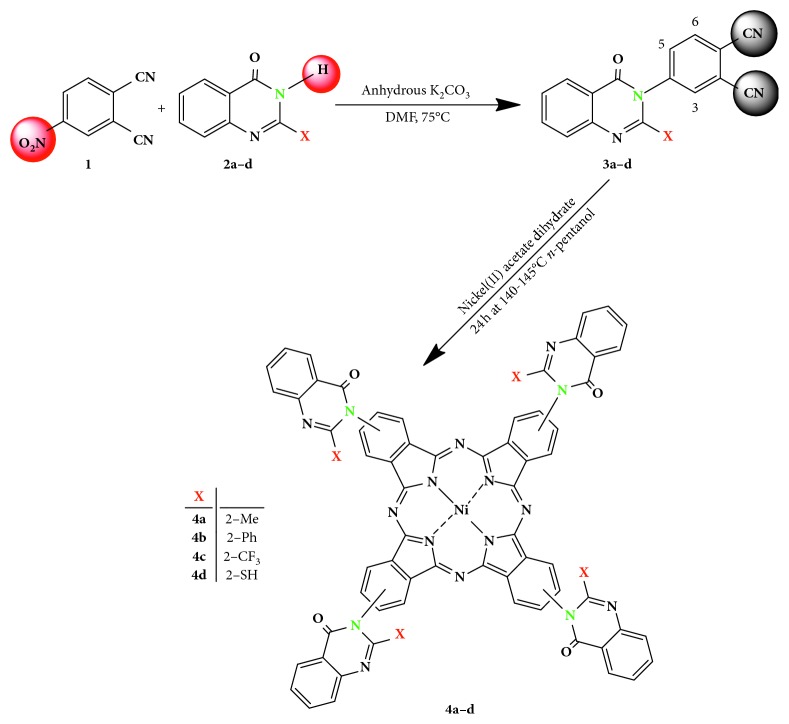
The synthetic pathway for the preparation of tetra quinazolinone nickel(II)phthalocyanine derivatives (**4a–d**).

Advanced synthetic procedure for the newly nickel(II)phthalocyanines (qz)_4_NiPcs **4a–d** substituted by 4(3H)-quinazolinone units has been described. Phthalonitrile derivatives **3a–d** were used by a two-step reaction procedure depicted in Figure 1. A nucleophilic ipso-nitro substitution reaction of 4-nitrophthalonitrile **1** was carried out with 4(3H)-quinazolinone derivatives **2a–d** in dry DMF for 24 h at 75–80°C. Following this, cyclotetramerization reaction of 4(3H)-quinazolinone-phthalonitriles precursors **3a–c** with Ni(II)acetate in the presence of DBU as organic base in *n*-pentanol for 30 h at 140–145°C afforded the corresponding (qz)_4_NiPcs (**4a–d**) with 71–74% yield. The desired phthalocyanines were separated chromatographically as a mixture of regioisomers from the reaction mixture (Figure 1).

The described synthetic method produced a mixture of four regioisomers with a 4(3H)-quinazolinone units at the 2 or 3 positions of each benzene ring in the (qz)_4_NiPc molecule. The formation of constitutional isomers [21, 22] and the high-dipole moment that results from the 4(3H)-quinazolinone units at the periphery positions leads to increase the solubility of the obtained products **4a–d**.

The ^1^H-NMR spectra of tetra-substituted nickel(II)phthalocyanines **4a–d** were obtained as expected. The ^1^H-NMR spectrum of (Me-qz)_4_NiPc **4a** indicated the methyl protons at *δ* = 1.30–1.6 ppm and the aromatic protons of Pc skeleton at 7.38 ppm. Also, the ^1^H-NMR spectrum of (Ph-qz)_4_NiPc **4b** indicated the aromatic protons of phenyl group at *δ* = 8.5–8.8 ppm. In case of (SH-qz)_4_NiPc **4d**, thiol proton appeared at *δ* = 3.38 ppm (see Section 2.2).

**Figure 2:**
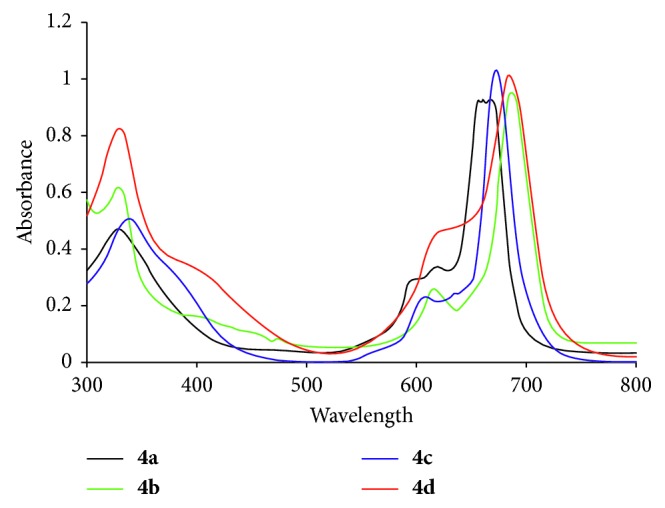
The absorption spectra of (qz)_4_NiPcs **4a–d** in DMF.

The electronic spectra of the studied nickel(II)phthalocyanines (qz)_4_NiPcs **4a–d** measured in DMF showed characteristic absorption bands in the Q band region at around 670, 682, 688, and 690 nm, respectively. The B-bands were observed at around 356, 352, 330, and 335 nm, respectively (Figure 2).

**Figure 3:**
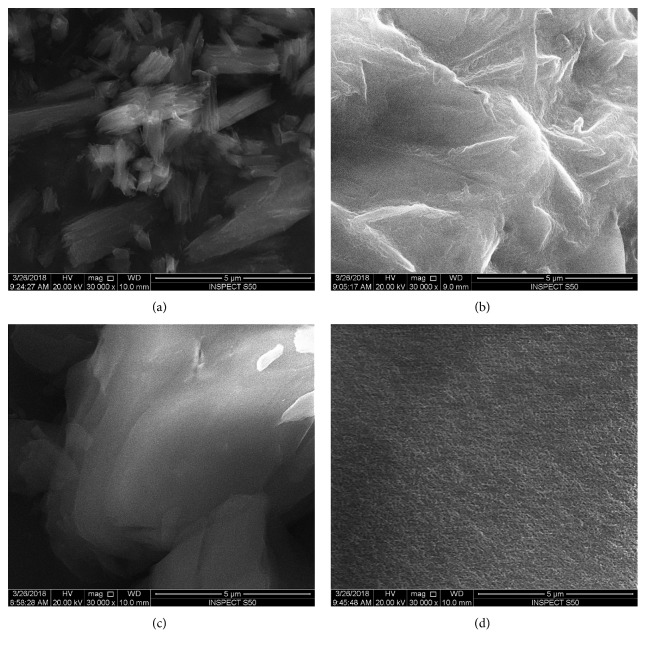
The surface morphology of the (qz)_4_Ni **Pcs 4a–d** samples prior to bacterial activities. SEM micrographs of (a) **4a**, (b) **4b**, (c) **4c**, and (d) **4d**. The samples show the different morphological features for different samples. All scale bars are 5 *μ*m.

SEM and TEM are commonly used tools to explore the microstructure of the materials.  Figure 3 shows the SEM micrographs of the phthalocyanines derivative products: control, additive of phenyl ring, trifluoro groups, and sulfur atoms. It was seen that the morphology of the product was completely altered with the addition of phthalocyanine derivatives. The pure phthalocyanines complex (Figure 3(a)) exhibited the fibrous-like structure compacted in bundle-shaped structure of varying sizes, sub-micrometer to few micrometer ranges. Phenyl and trifluoro groups-additive phthalocyanine derivatives (Figures 3(b) and 3(c)) showed the continuous but porous morphology, whereby fibrous bundles disappeared as seen in the pure phthalocyanines matrix. Phthalocyanines with sulfur also showed the porous morphology, but the surface of the complex was smooth compared to other specimens (Figure 3(d)).

**Figure 4:**
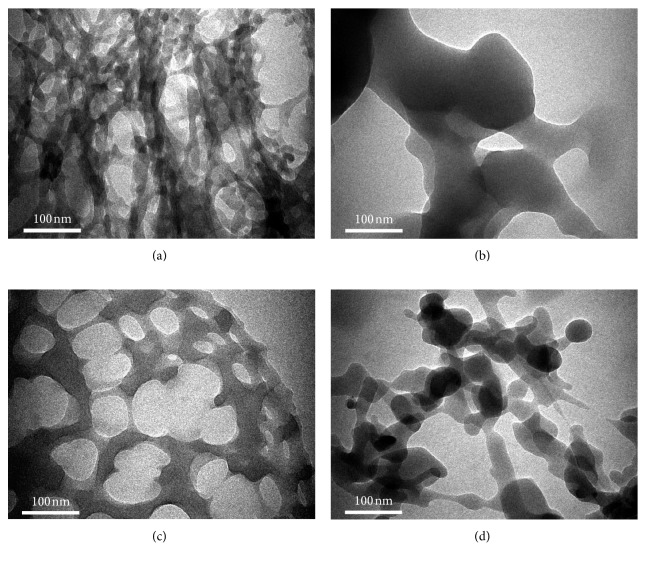
The morphology of the (qz)_4_NiPcs **4a–d** samples prior to bacterial activities. TEM representative images of (a) **4a**, (b) **4b**, (c) **4c**, and (d) **4d**. The samples show the different morphological features for different samples. All scale bars are 100 nm.

The structure of the phthalocyanines complexes was further investigated by TEM for high resolution (Figure 4). By TEM, it can be seen that all derivatives showed the porous structure of phthalocyanines but the porosity level and the morphology of the product vary for different complexes. The control specimen showed the sponge-like structure with pores from few nanometers to tens of nanometers (Figure 3(a)). The trifluoro-additive phthalocyanines product displayed the regular sized pore structure with pore size under 100 nm (Figure 4(c)). On the other hand, the sulfur-additive complex exhibited pores with material having a particle-alike structure, and the average size of the particles was estimated around 50 nm.

## 4. Discussion

In the present study, antibacterial properties of tetra-substituted 4(3H)-quinazolinone nickel (II)phthalocyanine derivatives against *Escherichia coli* ATCC 25922 have been evaluated by determining MICs and MBCs using agar dilution methods. The MICs and MBCs values of tetra-substituted 4(3H)-quinazolinone nickel(II)phthalocyanine derivatives **4a**, **4b**, **4c,** and **4d** are summarized in Table 1. The minimum MIC and MBC value recorded were 4 and 8 mg/ml for compound **4d**, whereas compounds **4c**, **4b**, and **4a** showed MICs values of 8, 8, and >16 and MBCs values of 16, 16, and >32 mg/ml, respectively. The antibacterial activities were found in the order of **4d** > **4c** > **4b** > **4a** against *Escherichia coli*, and the strongest antibacterial activity was achieved with compound **4d** (Table 1).

**Figure 5:**
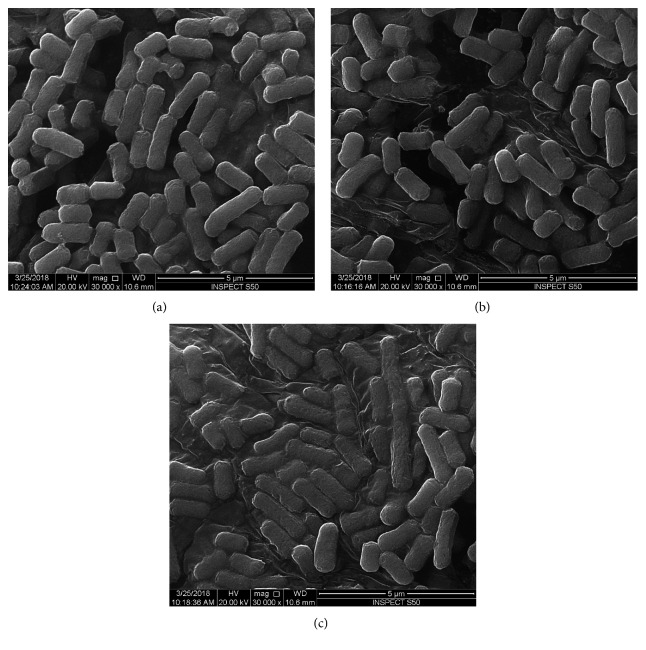
Scanning micrograph of *E. coli*: (a) *E. coli* control; (b, c) *E. coli* treated with 8 mg/ml of compound **4a**.

The morphological and structural changes in *Escherichia coli* cells caused by tetra-substituted 4(3H)-quinazolinone nickel (II)phthalocyanine derivatives were further investigated by SEM. The untreated (control) *Escherichia coli* cells were typically rod-shaped, intact, and normal having regular and smooth cell surface (Figure 5(a)).

**Figure 6:**
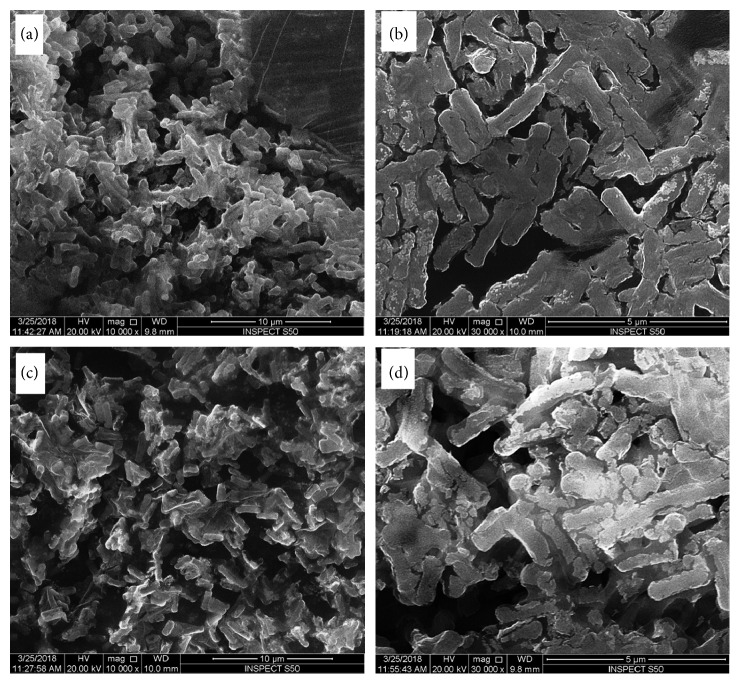
Antibacterial activity of compounds 4b (a, b) and 4c (c, d) against *E. coli* at 8 mg/ml. (a) and (c) are shown at lower magnification and (b) and (d) at higher magnification.

**Figure 7:**
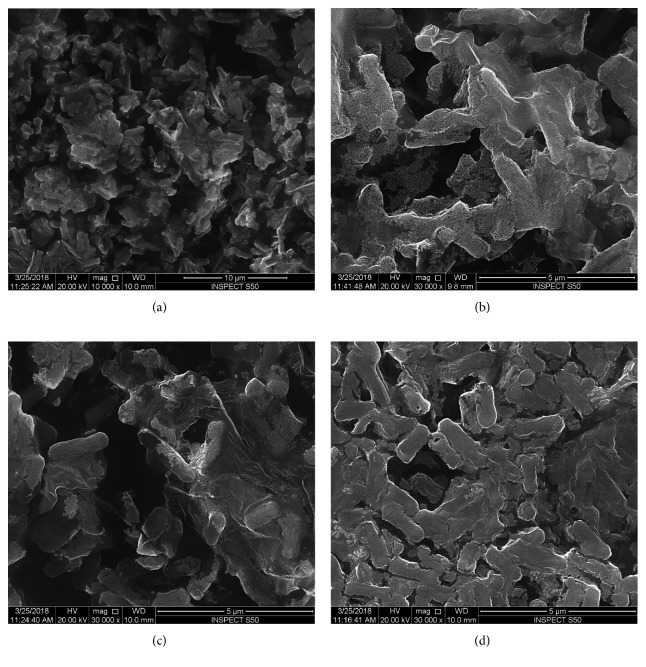
Scanning micrograph of *E. coli* treated with compound **4d** at a concentration of 1 mg/ml (a, b), 2 mg/ml (c), and 4 mg/ml (d). (a) is shown at lower magnification and (b)–(d) at higher magnification.

However, *Escherichia coli* cells treated with tetra-substituted 4(3H)-quinazolinone nickel(II)phthalocyanine derivatives (**4a**, **4b**, **4c** and **4d**) were not intact, i.e., the cells were abnormal in shape with irregular fragments appeared at the cell surfaces (Figures 5(b), 5(c), 6, and 7). The bacterial cells treated with compound **4a** were almost similar to that of control cells and no obvious alteration has been observed at a concentration of 8 mg/ml (Figure 5). The *Escherichia coli* cells treated with compounds **4b** and **4c** showed mild alternation in the morphology of cells at 4 mg/ml (Figure 6). However, *E. coli* cells treated with compound **4d** show significant alternation at a concentration of 4 mg/ml (Figure 7(b)).

Further, it has been observed that as the concentration of compound **4d** increases, it was noticed that the *Escherichia coli* cells were severely damaged because of formation of pits, indentation, deformation, and distortion of cell wall and membrane, indicating significant loss of the integrity of the cell membrane that may possibly lead to bacterial cell death (Figures 6(b)–6(d)). From the results, we suggested that the attachments of tetra-substituted 4(3H)-quinazolinone nickel(II)phthalocyanine derivatives to bacterial cell surface may play an important role in achieving good bactericidal activity.

There is very few information available in the literature regarding the mode of action of phthalocyanine derivatives against bacteria, but previous report on phthalocyanine derivatives suggested that singlet oxygen formed and ROS generated by phthalocyanine derivatives possibly interact with bacterial cell membrane that may damage the membrane integrity due to increased cell permeability and leakage of the intracellular materials [1, 4, 5].

## 5. Conclusion

The present study reports the successful synthesis of the title nickel(II)phthalocyanine (qz)_4_NiPcs **4a–d** in good yields. The results indicated that the studied (qz)_4_NiPcs **4a–d** possessed an activity against *Escherichia coli*. A preliminary study of the structure-activity relationship revealed that electronic factors in the 4(3H)-quinazolinone moiety that attached to the pc molecule has a great effect on the antibacterial activity of these nickel(II)phthalocyanines. The antibacterial activities were found in the order of **4d** > **4c** > **4b** > **4a** against *Escherichia coli*, and the strongest antibacterial activity was achieved with compound **4d**.

## Figures and Tables

**Table 1:** Minimum inhibitory concentration (MIC) and minimum bactericidal concentration (MBC) of (qz)_4_NiPcs **4a–d** against *E. coli*.

Compounds	MIC (mg/ml)	MBC (mg/ml)
**4a**	>16	>32
**4b**	8	16
**4c**	8	16
**4d**	4	8

## Data Availability

The data used to support the findings of this study are included within the article.

## Abbreviations

NiPcs: Nickel(II)phthalocyanines
(qz)_4_NiPcs: Tetra-substituted 4(3H)-quinazolinone nickel(II)phthalocyanines
*E. coli*: *Escherichia coli*.

## References

[1] Mikula P., Kalhotka L., Jancula D. (2014). Evaluation of antibacterial properties of novel phthalocyanines against *Escherichia coli*—comparison of analytical methods. *Journal Photochemistry and Photobiology B: Biololgy*.

[2] Jolanta D., Wojciech S., Tomasz K., Paulina S.-M. (2017). Antimicrobial and anticancer photodynamic activity of a phthalocyanine photosensitizer with N-methyl morpholiniumethoxy substituents in nonperipheral positions. *Journal Inorganic Biochemistry*.

[3] Canan U., Naciye D., Yetkin Ö., Burcu Turanl Y. (2018). A novel of PEG-conjugated phthalocyanine and evaluation of its photocytotoxicity and antibacterial properties for photodynamic therapy. *Journal Porphyrins and Phthalocyanines*.

[4] Rapulenyane N., Antunes E., Nyokong T. (2013). A study of the photophysicochemical and antimicrobial properties of two zinc phthalocyanine–silver nanoparticle conjugates. *New Journal of Chemistry*.

[5] Li M., Mai B., Wang A. (2017). Photodynamic antimicrobial chemotherapy with cationic phthalocyanines against *Escherichia coli* planktonic and biofilm cultures. *RSC Advances*.

[6] Chen Z., Zhou S., Chen J. (2014). An effective zinc phthalocyanine derivative for photodynamic antimicrobial chemotherapy. *Journal of Luminescence*.

[7] Dimaano M., Rozario C., Nerandzic M., Donskey C., Lam M., Baron E. (2015). The photodynamic antibacterial effects of silicon phthalocyanine (Pc) 4. *International Journal of Molecular Sciences*.

[8] Hemalatha K., Girija K. (2011). Synthesis of some novel 2,3 disubstituted quinozolinone derivatives as analgesic and antinflammatory agents. *International Journal Pharmaceutical Science*.

[9] Hanan G., Nagwa A.-G., Safinaz A. (2008). Synthesis and anticonvulsant activity of some quinazolin-4-(3H)-one derivatives. *Molecules*.

[10] Rudolph J., Esler W. P., O’Connor S. (2007). Quinazolinone derivatives as orally available ghrelin receptor antagonists for the treatment of diabetes and obesity. *Journal of Medicinal Chemistry*.

[11] Leila H., Alireza A., Mohsen R., Hamid S., Khajouei M. (2017). Synthesis and cytotoxic evaluation of some new 3-(2-(2-phenylthiazol-4-yl) ethyl)-quinazolin-4(3H) one derivatives with potential anticancer effects. *Research Pharmaceutical Science*.

[12] Soňa J., Štefan S., Katarína Š. (2004). *In vitro* antibacterial activity of ten series of substituted quinazolines. *Biologia Bratislava*.

[13] Connolly D. J., Cusack D., O’Sullivan T. P., Guiry P. J. (2005). Synthesis of quinazolinones and quinazolines. *Tetrahedron*.

[14] Dinakaran M., Selvam P., DeClercq E., Sridhar S. K. (2003). Synthesis, antiviral and cytotoxic activity of 6-bromo-2,3-disubstituted-4(3H)-quinazolinones. *Biological & Pharmaceutical Bulletin*.

[15] Kamal A., Vijaya Bharathi E., Janaki Ramaiah M. (2010). Quinazolinone linked pyrrolo[2,1-c][1,4]benzodiazepine (PBD) conjugates: design, synthesis and biological evaluation as potential anticancer agents. *Bioorganic & Medicinal Chemistry*.

[16] Youssef T. E., Hanack M. (2005). Asymmetrically fused heterocyclic phthalocyaninato nickel(II) adducts: synthesis and characterization. *Journal of Porphyrins and Phthalocyanines*.

[17] Youssef T. E., ALhamed Y., AL-Sharani S. (2014). Antitumor activity of tetra-substituted zinc phthalocyanines containing 4(3H)-quinazolinone derivatives. *Revista de Chimie (Bucharest)*.

[18] Al Jameel S. S., Youssef T. E. (2018). Investigations on the antitumor activity of classical trifluoro-substituted zinc phthalocyanines derivatives. *World Journal Microbiology and Biotechnology*.

[19] Ansari M. A., Khan H. M., Khan A. A., Sultan A., Azam A. (2012). Synthesis and characterization of the antibacterial potential of ZnO nanoparticles against extended-spectrum *β*-lactamases-producing *Escherichia coli* and *Klebsiella pneumoniae* isolated from a tertiary care hospital of North India. *Applied Microbiology and Biotechnology*.

[20] Essa A. M. M., Al Abboud M. A., Khatib S. I. (2018). Metal transformation as a strategy for bacterial detoxification of heavy metals. *Journal of Basic Microbiology*.

[21] Youssef T. E., Hanack M. (2002). Synthesis and characterization of unsymmetrically substituted dienophilic nickel phthalocyanines for Diels-Alder reactions. *Journal of Porphyrins and Phthalocyanines*.

[22] Helmut B., Venkata K., Youssef T. E., Hanack M. (2011). Structural investigations of hexadecafluoro(phthalocyaninato)ruthenium(II) F_16_PcRu with EXAFS spectroscopy. *Journal Porphyrins and Phthalocyanine*.

